# Pro-apoptotic and cytotoxic effects of enriched fraction of *Elytranthe parasitica* (L.) Danser against HepG2 Hepatocellular carcinoma

**DOI:** 10.1186/s12906-016-1395-3

**Published:** 2016-10-26

**Authors:** Nimmy Kumar, Subhankar Biswas, Asha Elizabeth Mathew, Subin Varghese, Jessy Elizabeth Mathew, K. Nandakumar, Jesil Mathew Aranjani, Richard Lobo

**Affiliations:** 1 Department of Pharmacognosy, Manipal College of Pharmaceutical Sciences, Manipal University, Manipal, 576 104 Karnataka India; 2 Department of Pharmacology, Manipal College of Pharmaceutical Sciences, Manipal University, Manipal, 576 104 Karnataka India; 3 Department of Pharmaceutical Chemistry, Manipal College of Pharmaceutical Sciences, Manipal University, Manipal, 576 104 Karnataka India; 4 Department of Pharmaceutical Biotechnology, Manipal College of Pharmaceutical Sciences, Manipal University, Manipal, 576 104 Karnataka India

**Keywords:** Anti-proliferative, Hepatocellular carcinoma, HepG2, *Elytranthe parasitica*, Gallic acid, Apoptosis, Phytochemical

## Abstract

**Background:**

Hepatocellular carcinoma (HCC), the most common type of liver cancer accounts for more than one million deaths worldwide. Current treatment modality for HCC is marginally effective. Plants belonging to Mistletoe family (Loranthaceae) have been used in chemotherapy for many years. The present study was aimed at exploring the anti-proliferative, pro-oxidant and pro-apoptotic potential of stem of *Elytranthe parasitica* (L.) Danser (EP), a parasitic shrub belonging to Loranthaceae.

**Methods:**

*Elytranthe parasitica* (L.) Danser, a climbing parasitic shrub was investigated for its cytotoxic activity against HepG2, a hepatocellular carcinoma cell line by Sulforhodamine B (SRB) assay. Further, pro-oxidant activity of EP extract/fractions was studied using copper phenanthroline assay. To understand the mechanism of cell death, the pro-apoptotic effects of Hep-G2 cells treated with EP extract/fractions were visualized by dual staining using acridine orange and ethidium bromide, a morphological marker of apoptosis. Phytochemical profiling of EP was explored by estimating the phenol, flavonoid and tannin content in its various fractions and extract. The occurrence of gallic acid, a principal polyphenol in EP extract and fractions was detected and further quantified using HPTLC (High Performance Thin Layer Chromatography) fingerprinting.

**Result:**

Active fraction of *Elytranthe parasitica,* EP.DEE exhibited potent cytotoxic activity in a dose dependent manner against HepG2 hepatocellular carcinoma cell line with an IC50 of 56.7 ± 7.8 μg/mL. Dual staining with acridine orange and ethidium bromide revealed that HepG2 cells treated with EP active fractions underwent cell death chiefly by apoptosis. Highest phenol, flavonoid and tannin content were observed in active fractions, EP.EA (Ethyl acetate fraction) and EP.DEE (Diethyl ether fraction). Gallic acid was identified and quantified in EP extract and active fractions, EP.DEE and EP.EA.

**Conclusion:**

Our findings indicate EP active fraction could be a promising contender in the treatment of hepatocellular carcinoma.

## Background

Hepatocellular carcinoma (HCC) is the most frequent and fatal type of liver cancer – a cancer that arises when a cell in the liver becomes genetically mutated allowing it to grow relentlessly [1]. The Cancer Statistics Report 2016 has recounted a higher incidence and mortality rate chiefly due to cancer of the liver [2]. With approximately five lakh new cases being diagnosed every year, HCC has become the fifth most common cause of cancer worldwide [3]. In India, death due to HCC has been on a rise in the recent years with a mortality rate of 6.8 per 100, 000 in men and 5.1 per 100,000 in women [4]. More often, patients diagnosed with HCC have very low survival rate, with merely 17.2 % of them surviving for 5 years or more after diagnosis [5]. Common treatment strategies for HCC include hepatectomy, liver transplantation, radiofrequency ablation (RFA), radiotherapy and chemotherapy [6]. Standard chemotherapy treatment involves administration of Sorafenib or Nexavar, a MAP Kinase inhibitor. Patients on Nexavar generally encounter adverse drug effects such as diarrhea, hand-foot skin disease, fatigue, anorexia and alopecia [7]. Medicinal plants and natural products, which have considerable chemotherapeutic value and comparatively fewer side effects [8–10], are increasingly being studied for treating cancer.

*Elytranthe parasitica* (L.) Danser [Synonym: *Macrosolen parasitica* (L.) Danser] is a perennial climbing woody parasitic shrub that belongs to Loranthaceae, the largest mistletoe family [11]. Known locally as Bandanekke or Baranike in Karnataka [12], this plant grows widely in the Western Ghats of India on peepal, mango, neem and jackfruit trees [13]. Mistletoes have a strong ethnopharmacological track record in the treatment of cancer [14, 15]. A primary example of this is *Viscum Album* or European mistletoe, that contains numerous chemopreventive and cytotoxic compounds such as lectins, viscotoxins and flavonoids [16]. Due to its powerful immune-modulating properties [17], formulations of mistletoe extracts such as Iscador, Helixor, Eurixor, Isorel have been used in oncology for many years [18]. Plants belonging to the Genus Elytranthe have shown substantial antitumor potential in previous studies [19, 20]. Earlier investigations on *Elytranthe parasitica* have focussed on their anti-tumour potential in Ehrlich Ascites Carcinoma model in Swiss albino mice [21] and against human breast adenocarcinoma cell line, MCF-7 [22]. We believe this is the first report exploring the cytotoxic effects of EP stem extract/fractions on HepG2 hepatocellular carcinoma cell line.

## Methods

### Chemicals and reagents

Petroleum ether, diethyl ether, butanol and toluene were purchased from Finar Limited, Ahmedabad, India. Ethanol was procured from Hayman Ltd, Essex, UK. Methanol, ethyl acetate and glacial acetic acid were purchased from Merck Specialties Pvt Ltd, Mumbai, India. Dulbecco’s Modified Eagle’s medium, Fetal Bovine Serum, Gentamycin, Sulforhodamine B (SRB), acridine orange, ethidium bromide, Folin Dennis reagent and gallic acid were obtained from Sigma Aldrich, St Louis, USA. Tris base and trypsin-EDTA were purchased from HiMedia Laboratories Pvt. Ltd, Mumbai, India. Trichloroacetic acid and sodium carbonate was purchased from Nice Chemicals Pvt Ltd, Kochi. Tissue culture flasks, multi-well plates and petri plates were obtained from Sigma Aldrich, St Louis, USA.

### Plant extraction

Stem of *Elytranthe parasitica* (EP) was collected from Manipal, Karnataka, India. The plant was authenticated by Dr. Gopalakrishna Bhat, Professor and Head, Department of Botany, Poornapraja College, Udupi, Karnataka, India. A voucher specimen (PP 565) has been deposited in the museum of Department of Pharmacognosy, Manipal College of Pharmaceutical Sciences, Manipal for future reference. EP stem (5 kg) was shade-dried, coarsely powdered and extracted with methanol by Soxhlet extraction to obtain the methanol extract (EP.M). After completion of extraction, the extract was concentrated under reduced pressure and controlled temperature and stored in the desiccator until further use.

### Preparation of fractions

The methanol extract of stem (200 g) of EP was fractionated sequentially with solvents petroleum ether, diethyl ether, ethyl acetate, butanol and water [23] to obtain petroleum ether fraction (EP.PE), diethyl ether fraction (EP.DEE), ethyl acetate fraction (EP.EA), butanol fraction (EP.But) and aqueous fractions (EP.Aq) respectively. All fractions were concentrated on a rotary evaporator to complete dryness and their percentage yield was calculated. The fractions were stored at 4 °C until further use.

### Cell line and culture condition

Human hepatocellular carcinoma cell line (HepG2) and normal kidney epithelial cell line (Vero) were obtained from National Center for Cell Sciences (NCCS), Pune. The cells were cultured in DMEM (Dulbecco’s Modified Eagle’s medium) supplemented with 10 % FBS. The cells were maintained at a constant temperature of 37 °C and under humidified conditions (in 5 % CO_2_) in an incubator.

### Cytotoxicity screening by Sulforhodamine B assay (SRB)

Cytotoxicity of EP extract and fractions were determined by Sulforhodamine B (SRB) assay [24]. Briefly, exponentially growing HepG2 cells were seeded into 96-well plates (5000 cells/well in 100 μL of media) and incubated for 24 h. Prior to the experiment, the test compounds were prepared in DMSO and diluted with media serially to obtain appropriate concentrations. Cells were treated with the extracts and fractions at concentrations of 25, 50, 100 and 200 μg/mL and incubated for 48 h. HepG2 cells in control group were treated with media containing 0.25 % DMSO. All treatments were carried out in triplicate. After 48 h of incubation from treatment period, to each well 100 μL of 10 % ice –cold trichloroacetic acid was added gently. The plate was subsequently incubated for an hour at 4 °C. Following this, the plate was sharply flicked, washed four times with 200 μL of deionized water and air dried. The plate was stained by adding 100 μL Sulforhodamine B (0.057 % w/v in 1 % acetic acid) and kept for 30 min in the dark at room temperature. Subsequently, the unbound dye was rapidly removed by rinsing four times with 1 % w/v acetic acid. The plate was air dried and excess water was removed by tapping on a tissue paper. 100 μL of 10 mM unbuffered Tris base was added to each well to solubilize the dye. The plate was shaken in a gyratory mixer with agitation for 10 min. Finally, absorbance was measured using spectrophotometer at 570 nm followed by the calculation of percentage cell viability and IC_50_ values using the following formula: Percentage cell viability = 100 – [((A-B)/A) × 100], where A = Absorbance of cells treated with 0.25 % DMSO medium, B = Absorbance of cells treated with extracts. Doxorubicin and gallic acid were used as standards.

### Total pro-oxidant potential

The potential of EP extract/fractions to impede DNA degradation mediated by the hydroxyl radicals was evaluated using the copper-1, 10 - phenanthroline complex method [25, 26] with slight modification. Accordingly, 1.2 mL of the reaction mixture comprising 100 μL of 9 mM 1, 10-phenanthroline, 240 μL of 5 M CuCl_2_.2H_2_O, 100 μL of 1 mg/mL DNA, 460 μL of 100 mM KH_2_PO_4_– KOH buffer (pH 7.4) was prepared. To it, 100 μL of 2.88 mM ascorbic acid or 100 μL of EP extract/ fraction (at a concentrations of 0.1, 1, 5 and 10 mg/mL) were added. The reaction mixture was subsequently incubated for one hour at 37 °C for 60 min. Afterwards, the following reagents were added in a sequential order: 100 μL of 0.1 M EDTA, 1 % (w/v) Thiobarbituric acid (in 0.05 M NaOH) and 1 mL of 25 % (v/v) HCl. Subsequently, the test samples were mixed in a vortexer and heated on a water bath for an hour at 100 °C. They were consequently cooled by immersing into an ice-water bath. To each test sample, 1.5 mL of n-butanol was added and the combination was mixed in the vortexer. Following this, the absorbance of the upper n-butanol layer was recorded at 530 nm. All experiments were performed in triplicate and data was presented as mean absorbance values and mean % inhibitory capacity (*n* = 3). Ascorbic acid was used as reference standard. Reaction mixtures with no test samples/standard served as control (100 % TBARS).

### Visualization of apoptosis by dual AO/EB staining

A chief hallmark of cancer cells is their adeptness to evade apoptosis and in consequence grow incessantly. Staining techniques such as AO/EB staining can distinctly detect the mode of cell death i.e. if cells have undergone cell death by apoptosis or necrosis [27]. AO/EB staining was done as per the standardized protocol [28]. Briefly, HepG2 cells (50,000 cells/well) in exponential phase were seeded onto 6-well plates and kept for 24 h incubation. The cells were treated with the test samples and incubated for 48 h. Subsequently, media was removed and 1 mL of 70 % ice cold ethanol was added to each well. The plate was kept for 2–4 h at 4 °C. Following this, ethanol was removed from the plate surface and rinsed with Phosphate buffered saline (PBS). The cells were stained with 300 μL of acridine orange/ethidium bromide dye (1 mg/mL) for 20 min. Thereafter, the dye was washed with PBS two times. The cells were observed under fluorescent microscope (Zeiss, Germany) and morphology of cells was observed. Untreated HepG2 cells and Curcumin treated HepG2 cells served as the control and standard groups respectively. Viable and apoptotic cells were counted and represented as mean ± SEM.

### Phytochemical constitution of EP

#### Preliminary phytochemical screening and thin layer chromatographic studies

EP extract and fractions were screened for their phytoconstituents [29]. Further, thin layer chromatography was performed alongside reference standard gallic acid in Toluene: Ethyl acetate: Formic acid: Methanol (3:3:0.8:0.2) [30].

### Total phenolic content

Folin-Ciocalteu method was utilized to measure the total phenolic content of EP extract and fractions as per the standard protocol [31].

### Total flavonoid content

Aluminium chloride colorimetric method was followed to determine the total flavonoid content in EP extract and fractions [32].

### Total tannin content

Folin Denis method was employed to detect the presence of tannins in the investigational plant [33].

### High performance thin layer chromatography fingerprinting

Gallic acid was quantified in EP extract and fractions by HPTLC fingerprinting [30]. Briefly, test solutions of EP extract/fractions were prepared at a 2 mg/mL concentration in methanol. Gallic acid solution was prepared at a concentration of 100 μg/mL in methanol. HPTLC Silica gel 60 F254 plate (Merck Life Sciences Ltd Pvt, Mumbai) with a dimension of 20 cm x 10 cm served as the stationary phase. Mobile phase was prepared by saturating the 20 cm x 10 cm Twin trough chamber with 10 mL of Toluene: Ethyl acetate: Formic acid: Methanol (3:3:0.8:0.2) for twenty minutes. Using the Camag Linomat 5 sample applicator, outfitted with liquid nitrogen tank, 10 μL per sample/standard was sprayed in the form of bands onto the HPTLC plate at a distance of 1.5 mm from the plate base. Sample solutions were loaded onto the sample applicator with a 100 μL HPTLC syringe (Camag Linomat syringe 695.0014, Hamilton Bonaduz, Schweiz). The plate was air dried and developed in the Twin trough chamber up to a distance of 80 mm from the point of application. Using Camag TLC Scanner 3, the HPTLC plate was scanned densitometrically under a scanning speed of 20 mm/s at a single wavelength of 280 nm. Subsequently, UV absorption spectrum of bands corresponding to gallic acid was examined at the same wavelength.

### Statistical analysis

Results are expressed as Mean ± Standard Error of Mean (SEM). GraphPad Prism 5.0 software (GraphPad Software, Inc., California) was utilized for preparing graphs and for estimation of IC50 via the non linear regression equation. Statistical significance (*P* value) was ascertained by one-way ANOVA, followed by Tukey’s post hoc test of significance between different groups, where, *P* < 0.05 was estimated to be significant.

## Result and discussion

### Effects of EP extract/fraction on HepG2 and Vero cells

**Fig. 1:**
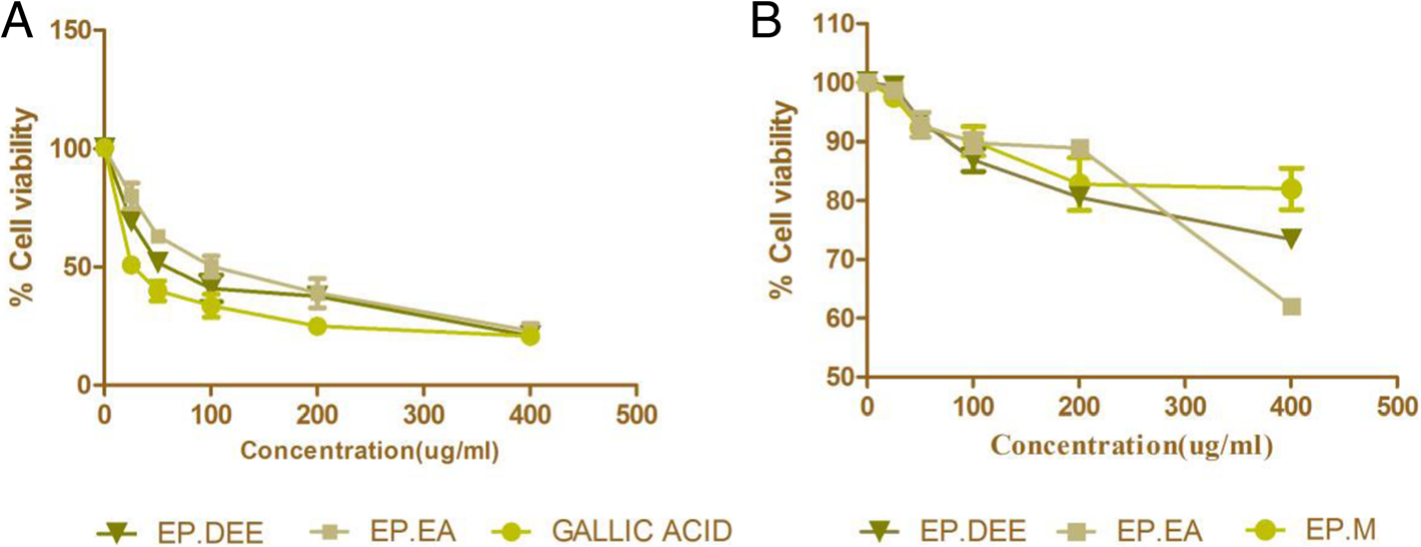
Effect of EP extract/fractions on cell viability and growth of (**a**) human HepG2 hepatocellular carcinoma cells and (**b**) Vero cell line. HepG2 and Vero cells were seeded onto 96 well plate at a density of 5,000 cells/well and subsequently treated with EP extract/fractions for 48 h. Data is expressed as IC50 (mean ± SD) of three individual experiments. Cell viability was estimated by Sulforhodamine B assay. EP. DEE- Diethyl ether fraction, EP.EA- Ethyl Acetate fraction, EP.M- Methanol extract

EP extract/fractions were tested against HepG2 hepatocellular carcinoma cell line and Vero (normal kidney epithelial) cells using SRB assay. SRB assay, which was originally, developed by Skehan et al in 1990 gives an accurate and reproducible measurement of the cellular protein content. It works primarily on the principle of the pink aminoxanthine SRB dye binding to the basic amino acid residues of the proteins present in the cell [34]. Of the tested samples, fractions EP.DEE and EP.EA were found to be most active against HepG2 cell line with an IC50 of 56.7 ± 7.8 μg/mL and 101.33 ± 5.3 μg/mL respectively (Fig. 1a). The active fractions also displayed concentration-dependent inhibition of cell growth (Table 1). EP.M showed moderate cytotoxicity against HepG2 carcinoma cell line (IC50 = 154.12 ± 2.6 μg/mL). The remaining fractions, EP.PE, EP.But and EP.Aq were unable to prevent cell proliferation at very high concentrations and were considered to be inactive. Doxorubicin, the standard used presented significantly high cytotoxicity with an IC50 of 1.9 ± 0.3 μM. Several studies have proven gallic acid, a predominant polyphenol to possess remarkable anti-cancer activity [35]. Gallic acid was identified in the EP extract and fractions by Thin Layer Chromatography. Due to its confirmed presence in the plant, we employed gallic acid as the second standard in the assay to study if the cytotoxic activity of EP could partially be attributed to the presence of gallic acid. We observed gallic acid to exhibit potent cytotoxic activity against HepG2 cell line in a dose-dependent manner (IC50 = 31.05 ± 6.2 μg/mL). Our results concurred with previous studies [36, 37] which investigated the cytotoxic activity of gallic acid against HepG2 cell line. At the highest tested concentration (400 μg/mL), EP fractions and extract did not exert cytotoxic effects on Vero cells (Fig. 1b).

**Fig. 2:**
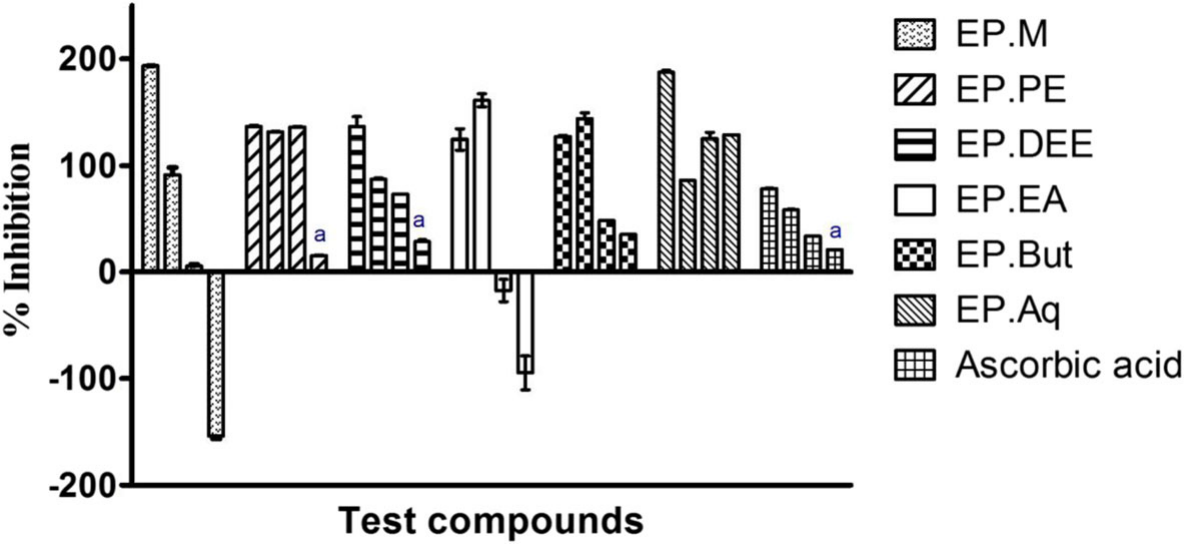
% Inhibitory effect of EP extract/fractions in hydroxyl radical mediated DNA degradation assay. The test compounds were screened at concentrations of 10, 100, 500 and 1000 μg/mL (from left to right). Ascorbic acid was employed as standard at the same concentrations. Results are expressed as % inhibitory capacity (mean ± SD) of three individual experiments. Statistical Analysis was performed by 2 way ANOVA followed by Bonferroni’s post hoc test of significance. Columns with similar superscripts did not differ significantly (*P* > 0.05). EP. M- Methanol extract, EP.PE -Petroleum ether fraction, EP. DEE- Diethyl ether fraction, EP.EA- Ethyl Acetate fraction, EP. But - Butanol fraction, EP.Aq- Aqueous fraction

**Table 1:** Effect of EP extract/fractions on % cell viability of HepG2 carcinoma cell line by SRB assay

Concentration (μg/mL)	EP.M	EP.PE	EP.DEE	EP.EA	EP.But	EP.Aq	Gallic acid
0	100.00 ± 0.33	100.00 ± 0.33	100.00 ± 0.33	100.00 ± 0.33	100.30 ± 0.33	100.30 ± 0.30	100.30 ± 0.30
25	96.80 ± 2.40	99.20 ± 5.60	59.30 ± 6.70	80.00 ± 0.50	99.60 ± 5.50	99.80 ± 4.50	53.80 ± 5.66
50	72.30 ± 5.50	99.00 ± 2.10	53.70 ± 11.90	63.10 ± 1.22	99.30 ± 3.80	99.10 ± 2.30	42.90 ± 9.20
100	69.00 ± 3.70	98.40 ± 0.40	40.90 ± 5.60	50.20 ± 4.40	94.80 ± 3.10	98.30 ± 1.50	33.50 ± 4.90
200	42.50 ± 2.10	86.00 ± 9.10	36.60 ± 3.98	38.90 ± 6.20	91.60 ± 7.80	93.00 ± 4.10	24.90 ± 6.89
400	16.40 ± 7.20	83.00 ± 9.00	20.90 ± 7.10	22.90 ± 3.10	72.40 ± 5.40	86.00 ± 3.20	20.70 ± 4.62
IC50	154.10 ± 2.60^c^	> 200	56.70 ± 7.80^a^	101.30 ± 5.3^b^	> 200	> 200	31.00 ± 6.21^a^

Results are expressed as mean ± SEM (of three observations) and analyzed using one-way ANOVA followed by Tukey’s post hoc test of significance [where different alphabets ^a-c^denote significant difference (*p* <0.05)]. EP.*M* Methanol extract, EP.*PE* Petroleum ether fraction, EP.*DEE* Diethyl ether fraction, EP.*EA* Ethyl Acetate fraction, EP.*But* Butanol fraction, EP.*Aq* Aqueous fraction

### Pro-oxidant potential of EP extract/fractions

Several plants enriched with polyphenols are known to possess dual anti-oxidant effects at low concentrations and pro-oxidant effects against DNA at higher concentrations [38]. EP extract/fractions were assessed for their pro-oxidant and anti-oxidant effects using copper phenanthroline induced DNA damage assay. In control group, DNA was heated with the (1, 10- phenanthroline) Cu (II) complex which resulted in the production of TBARS that is evidenced by the increase in absorbance. Ascorbic acid at all concentrations (10, 100, 500 and 1000 μg/mL) considerably augmented the quantity of hydroxyl radical-generated TBARS from the basal level. At 10 μg/mL, EP.M did not increase absorbance above the basal level; however at 100, 500 and 1000 μg/mL, the absorbance was higher than control. EP.PE exhibited high absorbance at the highest tested concentration of 1000 μg/mL. There was increased absorbance in EP.DEE, EP.EA and EP.But fraction at higher concentrations of 500 and 1000 μg/mL. EP.Aq exhibited lesser absorbance than control at all concentrations (Table 2). Clearly, EP.M, EP.DEE, EP.EA and EP.But have anti-oxidant effects at lower concentrations of 10 and 100 μg/mL and pro-oxidant effects at 500 and 1000 μg/mL (Fig. 2).

**Table 2:** Effect of EP extract/fraction on copper - 1, 10 - phenanthroline dependent DNA damage

Concentration (μg/mL)	Extent of DNA damage (A_530nm_)						
Test compounds	EP.M	EP.PE	EP.DEE	EP.EA	EP.But	EP.Aq	Ascorbic acid
10	0.04 ± 0.001	0.04 ± 0.008	0.04 ± 0.004	0.04 ± 0.002	0.04 ± 0.004	0.03 ± 0.004	0.08 ± 0.004
100	0.06 ± 0.005	0.04 ± 0.004	0.07 ± 0.004	0.04 ± 0.001	0.04 ± 0.002	0.07 ± 0.002	0.10 ± 0.009
500	0.12 ± 0.001	0.04 ± 0.007	0.08 ± 0.002	0.13 ± 0.002	0.13 ± 0.004	0.05 ± .0.001	0.18 ± 0.007
1000	0.22 ± 0.002	0.11 ± 0.001	0.10 ± 0.002	0.18 ± 0.003	0.17 ± 0.008	0.04 ± 0.001	0.30 ± 0.007
Control	0.06 ± 0.003						

Values are expressed as mean ± SEM (of three observations). EP.*M* Methanol extract, EP.*PE* Petroleum ether fraction, EP.*DEE* Diethyl ether fraction, EP.*EA* Ethyl Acetate fraction, EP.*But* Butanol fraction, EP.*Aq* Aqueous fraction

1, 10 copper phenanthroline assay is a frequently used assay to examine the pro-oxidative activity of extracts against DNA [26]. On addition of reagents and subsequent incubation, a complex called bis (1, 10 – phenanthroline copper) is formed which links to DNA and breaks down its strand; hydroxyl radical generation is primarily implicated in DNA damage [39]. However, if the cuprous ions are bound, DNA degradation does not occur. When reducing agent ascorbic acid (which is a proven pro-oxidant at higher concentration [40]) is added to the complex, Cu(II) in the complex gets converted to Cu(I) and hence, there is additional production of hydroxyl radicals that breaks down DNA into TBARS (Thiobarbituric Acid Reactive Substance). Hence, the level of TBARS formed in different extracts/fractions can indirectly reveal the quantity of hydroxyl radicals generated and give an estimation of the pro-oxidant or anti-oxidant activity of the extract/fractions at varying concentration. ROS (Reactive Oxygen Species) often personate a “double edged sword” in chemotherapy [41]. While in the initial stages of cancer, they may contribute to malignant transformation; in the more advanced stages of cancer, ROS can exert cytotoxic effect on the malignant cells. Normal cells may generally be spared from this fate as they have comparatively low basal levels of ROS. The ROS threshold level is therefore higher in cancer cells and once this level is reached, apoptosis is preferentially triggered in cancer cells alone, thereby preventing the metastasis of cancer to vital organs [42]. However, to evade apoptosis, cancer cells often adapt their anti-oxidant defense in response to the high oxidative stress and so become drug resistant [43]. Recently, several studies have investigated how the level of pro-oxidants agents can be manipulated by redox modulation and ROS mediated mechanisms to exclusively target cancer cells to go into a state of apoptosis and prevent cancer cells from becoming drug resistant [44, 45]. However, to wholly exploit the ROS-mediated cell-death mechanism as a treatment strategy against anticancer resistant cancer cells, combinations of ROS generating compounds with agents that suppress the cellular antioxidant capacity could be employed against cancer cells [42]. Our preliminary findings from copper phenanthroline mediated DNA damage assay indicate that EP fractions (EP.DEE, EP.EA, EP.But) and EP.M exhibit substantial ROS generating capacity at higher concentrations.

### Pro-apoptotic effects of EP active fraction

Apoptosis is a highly synchronized process of cell death, wherein cell fragments shrink to form ‘apoptotic bodies’ that are eventually phagocytosed by the nearby cells. However, due to the numerous mutations they harbor, cancer cells are adept at evading apoptotic process to thwart their self-destruction [46]. Cytotoxic compounds which selectively initiate apoptosis in cancer cells and thus slow down their growth are considered to be potential candidates for chemotherapy [47]. Acridine orange (AO) and ethidium bromide (EB), two fluorescent DNA binding dyes (which differentially infiltrate the cells emitting green and red fluorescence respectively) are used in AO/EB staining to detect apoptosis-related transformations in the cell membrane during apoptosis. *Per se*, the technique distinguishes between viable, early apoptotic, late apoptotic and necrotic cells [27]. Both AO and EB intercalate with DNA and stain the cell nuclei differently. While AO is cell permeable and stains the nuclei of viable cells and early apoptotic cells green, EB is impermeable to cells with an intact cell membrane. EB solely enters cells with damaged and compromised cell membrane and stain the nuclei orange [48].

**Fig. 3:**
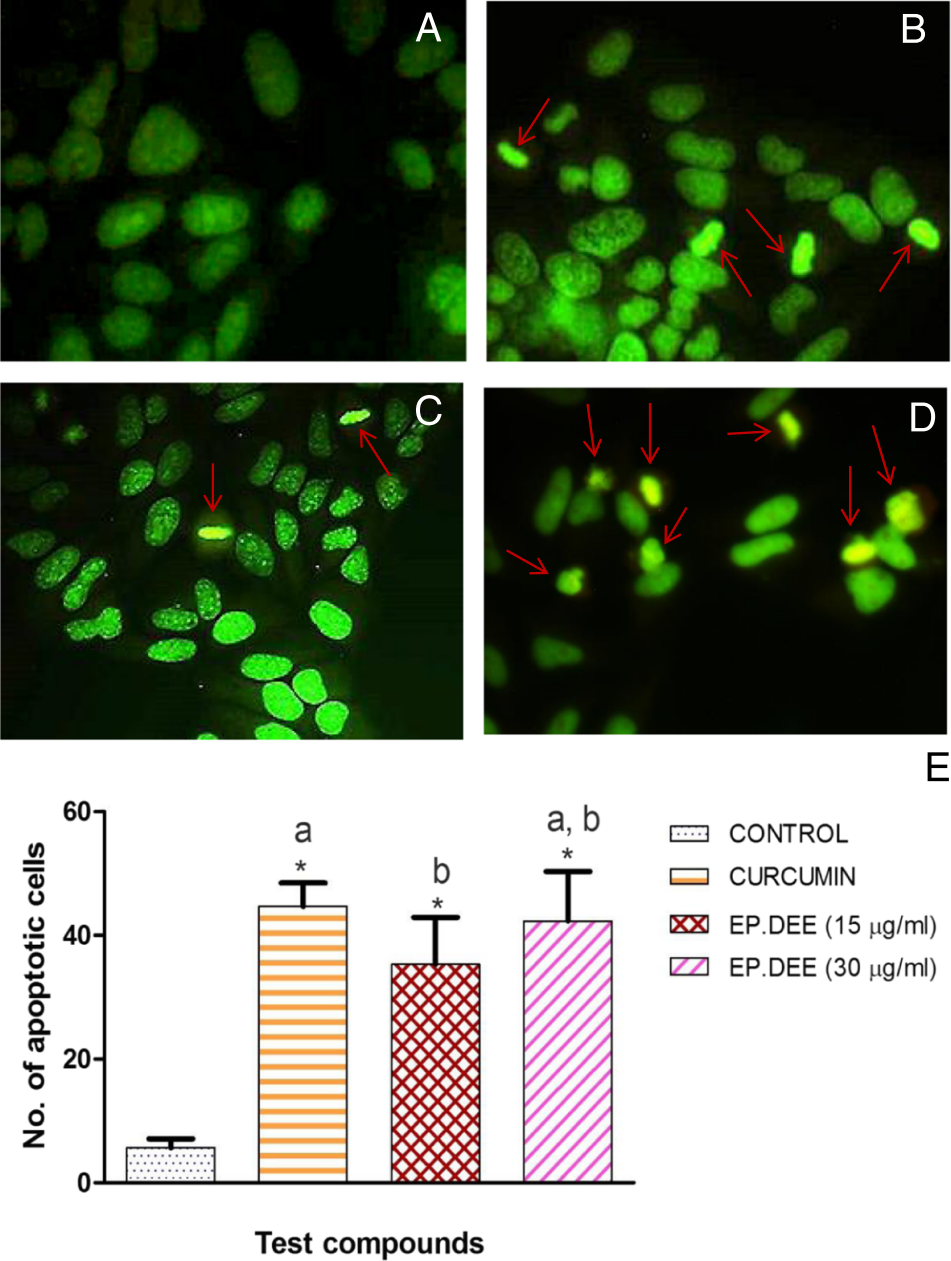
Visualization of apoptosis in HepG2 hepatocellular carcinoma cells after treatment (AO/EB dual staining) HepG2 cells were seeded onto 6 well plates at a density of 50,000 cells/well, treated with test compounds and stained with AO/EB dye after 48 h of treatment. The treatment groups are as follows: (**a**) Control or untreated HepG2 cells (**b**) Curcumin reference group (4 μg/mL) (**c**) EP. DEE (15 μg/mL) (**d**) EP. DEE (30 μg/mL). Arrows in the figure are directed towards the apoptotic bodies, that exhibited bright yellowish-green fluorescence. Condensed chromatin was observed in all treated groups. Control group cells showed normal morphological features with undamaged cell membrane. **e** Graph depicting number of apoptotic cells in different treatment groups. *P value > 0.05 when compared to control group. Columns with different superscripts differ significantly (*P* > 0.05). EP. DEE- Diethyl ether fraction

HepG2 cells treated with active EP fraction, EP.DEE underwent considerable cytoplasmic shrinkage compared to untreated cells (Fig. 3). Other distinctive traits of apoptosis such as condensed chromatin and appearance of apoptotic bodies were spotted in EP.DEE treated group. Apoptotic cells were identified by their bright yellowish-green fluorescence and typical crescent- like appearance on one side of the cell. HepG2 cells treated with lower concentration of active fraction appeared bright yellowish-green indicating the cells to be in early apoptotic stage of cell death. Cells subjected to higher concentration of active fraction had more number of bright yellowish-green apoptotic cells. Untreated cells (in control group) were consistently green in color, with normal morphological features and undamaged cell membrane. Curcumin-treated cells were stained a bright yellowish green also indicating apoptotic death of cells. Treatment with high concentrations of active fraction did not cause necrosis; hence mode of cell death of treated HepG2 cells was ascertained to be via apoptosis.

### Phytochemical profiling of EP

#### Phytochemical screening results

EP extract and fractions were screened for the presence of secondary metabolites. EP.M, EP.PE and EP.DEE gave a positive test for Liebermann-Burchard and Salkowski test revealing phytosterols. EP.DEE, EP.EA and EP.But gave an instant crimson red color in Shinoda’s test, implying presence of flavonoids in these fractions. EP.M, EP.DEE, EP.EA, EP.But and EP.Aq tested positive (greenish-black color) for Ferric chloride test for phenols and tannins. Saponins and caarbohydrates were detected in EP.M extract and EP.Aq fraction.

### Phenol, flavonoid and tannin content

Polyphenols are a diverse group of phytoconstituents comprising anthocyanins, isoflavonones, flavonoids, tannins and catechins. Myriad of health benefits have been attributed to this group of phytoconstituents largely due to their key role in protecting the human body against multiple diseases. In the past, several epidemiological studies have investigated the chemopreventive effects of polyphenols against various malignancies [49]. Flavonoids and tannins, two sub types of polyphenolic compounds have been widely acknowledged to possess remarkable anticancer potential. Since, polyphenols are credited with such remarkable anticancer properties; we endeavored to estimate the total phenolic, flavonoid and tannin content in the plant extract/fractions to explore any relation between their occurrence and the chemotherapeutic potential of the plant.

Gallic acid was used as the reference standard for determining total phenolic content by Folin-Ciocalteau method and a calibration curve was plotted (R^2^ = 0.9981). EP.EA and EP.DEE fractions were found to be highly enriched with phenolic compounds with an estimated phenolic content of 91.01 ± 2.68 and 72.48 ± 2.43 mg GAE/g of plant fraction respectively. For estimation of flavonoid content by aluminium chloride colorimetrc method, quercetin was employed as the reference standard and used in concentrations ranging from 15.625 to 500 μg/mL to plot a calibration curve (R^2^ = 0.999). Highest flavonoid content was observed in EP.DEE (14.41 ± 0.52 mg QE/g of plant fraction). EP.EA also had sufficiently high flavonoid content (12.93 ± 0.65 mg QE/g of plant fraction). For tannin content estimation by Folin-Dennis method, gallic acid was used as the positive control. Calibration curve of gallic acid was plotted to estimate the tannin content in EP extract and fractions (R^2^ = 0.9995). The level of tannins in EP extract and fractions, estimated by Folin–Dennis method and expressed in Gallic acid Equivalent (GAE) were similar to the level of phenolics quantified by Folin-ciocalteau method. The highest content of tannin was found in fractions EP.EA (22.80 ± 0.19 mg GAE/g of plant fraction) and EP. DEE (17.10 ± 0.13 mg GAE/g of plant fraction). Sufficiently high tannin content was noted in the butanol fraction (13.57 ± 0.20 mg GAE/g of plant fraction). Table 3 lists the total phenol, flavonoid and tannin content in EP extract and fractions.

**Table 3:** Total phenol, flavonoid and tannin content in EP extract/fractions

S. No.	Extracts/fractions	Total phenol content (mg GAE/g of plant extract)	Total flavonoid content (mg QE/g of plant extract)	Total tannin content (mg GAE/ g of plant extract)
1.	EP.M	66.97 ± 4.78^b^	10.87 ± 0.85^b^	7.18 ± 0.12
2.	EP.PE	36.85 ± 1.05^d^	12.97 ± 1.55^b^	4.37 ± 0.15
3.	EP.DEE	84.38 ± 1.55^a, c^	15.72 ± 2.85^a,b^	17.10 ± 0.13^a^
4.	EP.EA	92.17 ± 4.48^a^	22.48 ± 1.51^a^	22.80 ± 0.19^a^
5.	EP.BUT	77.43 ± 6.25^b, c^	12.29 ± 5.32^b^	13.57 ± 0.20
6.	EP.AQ	31.56 ± 1.47^d^	2.29 ± 0.21	0.64 ± 0.09

Data is presented as mean ± SEM (*n* = 3); Equivalent. Results were analyzed by one way ANOVA followed by Tukey’s test. Values in the same column followed by a different superscript ^a-d^are significantly different (*P* < 0.05). *GAE* Gallic acid Equivalent, *QE* Quercetin, EP.*M* Methanol extract, EP.*PE* Petroleum ether fraction, EP.*DEE* Diethyl ether fraction, EP.*EA* Ethyl Acetate fraction, EP.*But* Butanol fraction, EP.*Aq* Aqueous fraction

### High performance thin layer chromatographic fingerprint profile

**Fig. 4:**
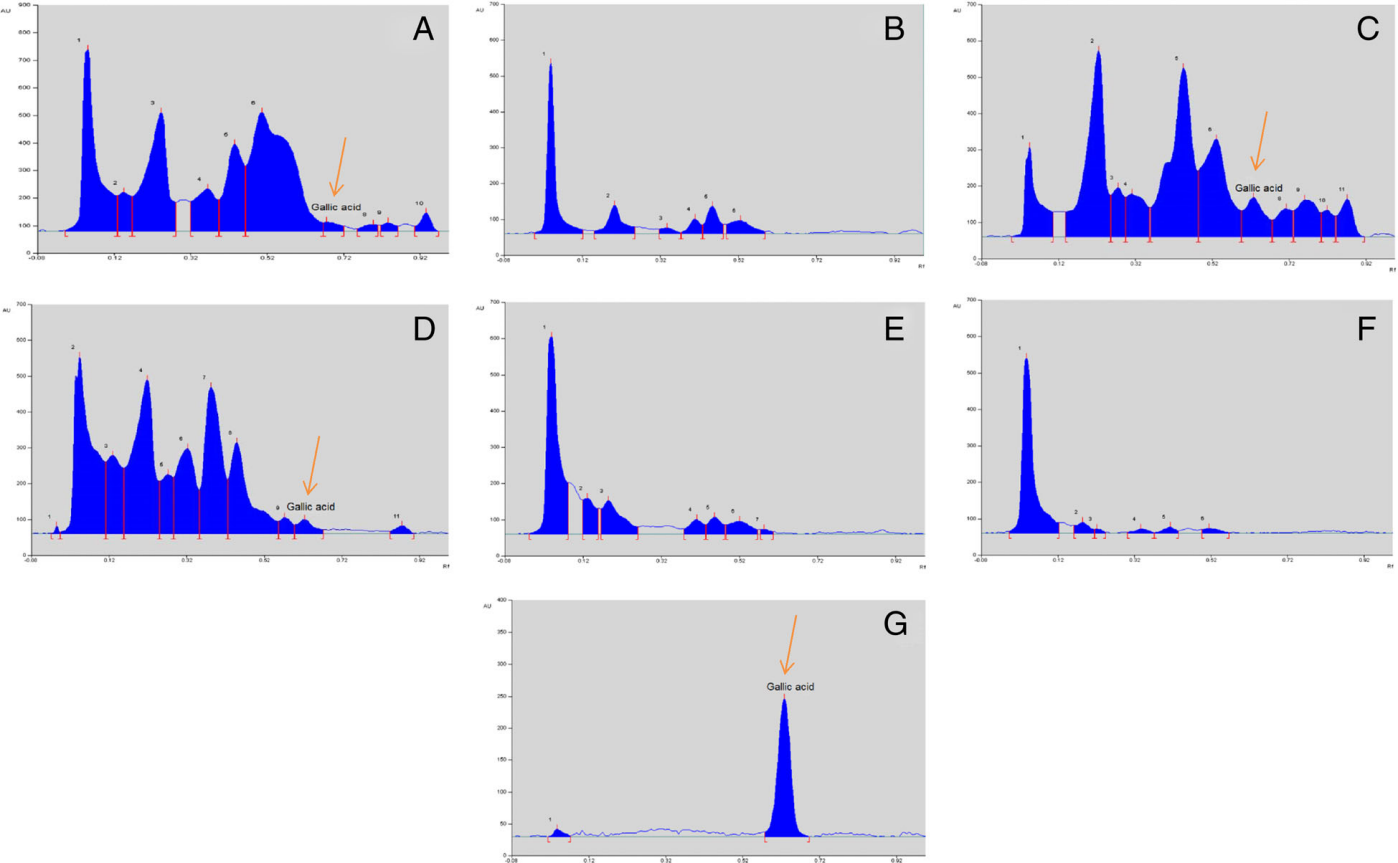
HPTLC fingerprint profile of EP extract and fractions (**a**) EP.M, Methanol extract (**b**) EP.PE, Petroleum ether fraction (**c**) EP. DEE, Diethyl ether fraction (**d**) EP.EA, Ethyl acetate fraction (**e**) EP. But, Butanol fraction (**f**) EP.Aq, Water fraction fraction (**g**) Gallic acid (reference standard)

**Fig. 5:**
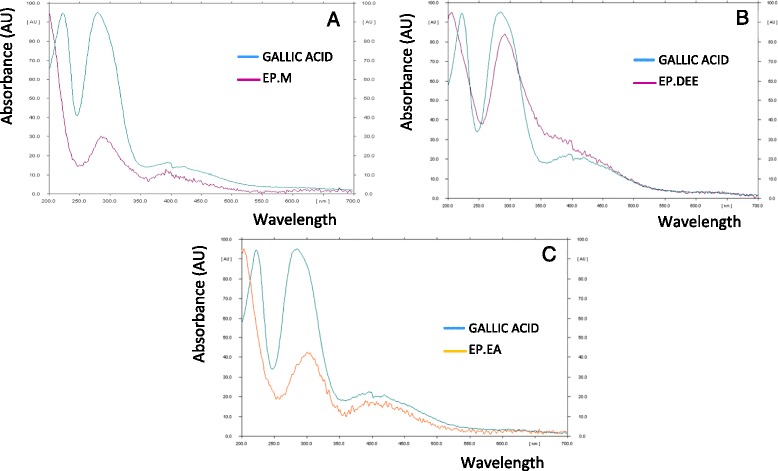
Overlay of UV absorption spectra of reference gallic acid and corresponding band in EP extract/fractions [Absorbance, AU vs. Wavelength, nm] (**a**) Gallic acid and EP.M extract (**b**) Gallic acid and EP. DEE (**c**) Gallic acid and EP.EA

High Performance Thin Layer Chromatography with gallic acid as reference standard afforded well resolved peaks in EP extract and fractions, with good separation of gallic acid from other phytoconstituents. Figure 4 depicts the HPTLC fingerprint profile of EP extract and fractions. Reference standard gallic acid eluted at Rf (Retention factor) of 0.63. Gallic acid was detected in EP extract and bioactive fractions, EP. DEE and EP.EA. EP.DEE, the active fraction gave mainly eleven peaks, of which gallic acid was peak no. 7 (Rf value - 0.63, 2.173 ± 0.053 %). EP.EA gave eleven peaks, of which gallic acid was peak no. 10 (Rf value - 0.63, 0.528 ± 0.01 %). EP methanol extract also contained gallic acid, however in comparatively less quantity (peak no 7, Rf value - 0.63, 0.293 ± 0.016 %). Gallic acid was not observed in other EP fractions EP PE, EP.But and EP.Aq. To confirm the presence of gallic acid in EP, the UV spectrum of gallic acid at 280 nm was compared with the corresponding peak (at Rf 0.63) in EP extract/fraction. The UV spectral characteristics matched, verifying the presence of gallic acid in EP.M, EP. DEE and EP.EA (Fig. 5).

It is widely accepted that the additive and synergistic anti-cancer effect of plant extracts are hugely due to the multiple bioactive substances present in them [49]. Often, individual components isolated from plant exert sufficiently lower activity than the combined plant extract. *Per se*, the anticancer activity of EP maybe primarily attributed to the synergistic activity of the plentiful phyto components present in the plant, one of which is gallic acid. Our research work indicates that gallic acid maybe one of the phytoconstituents that contributes to the overall anti-tumor potential of EP. Gallic acid, a principal polyphenolic molecule that occurs naturally in several plant based foods has been credited with remarkable anti-tumor activity [35]. Several studies have proven gallic acid to exhibit potent *in vitro* cytotoxic activity against various cancer cell lines of lung, cervix, liver etc [35]. Among the liver cancer cell lines, gallic acid induced apoptosis in SMMC-7721 hepatocellular carcinoma cell line via the mitochondrial mediated pathway by various mechanisms involving activation of pro-enzymes caspase 3 and caspase 9, induction of ROS pathway, inhibition of mitochondrial membrane potential (MMP) and upregulation of Bcl-2-like protein [36]. Moreover, gallic acid showed 3-fold higher sensitivity towards hepatic cancer cell lines HepG2 and SMMC-7721 when compared to normal hepatocyte cell line HL-7702 [36, 50]. The underlying Structure Activity Relationship (SAR) of gallic acid’s selective anticancer activity was due to its carboxyl group which differentiated between cancer and non-cancerous cells and so exclusively triggered apoptosis in cancer cells [51]. Several studies have also indicated gallic acid to possess potent pro-oxidant potential [52]. Hence, presence of gallic acid may partially be responsible for the cytotoxic and pro-oxidant potential of EP extract/fractions.

## Conclusion

EP.DEE, the active fraction was found to be highly toxic against HepG2 cell line and also exhibited high pro-oxidant potential akin to ascorbic acid. Phytochemical characterization studies in EP revealed presence of polyphenols in the plants with especially higher flavonoid, tannins and phenolic content in the active fractions EP. DEE and EP.EA. Gallic acid was identified and quantified in active fractions. Our findings further corroborate that EP.DEE caused cell death in HepG2 cells via apoptosis, signifying the fraction to be a promising contender in treating HCC. However, further studies to comprehend the underlying molecular mechanism for its anti-cancer activity are requisite for establishing its effectiveness in HCC therapy. Studies on identification and isolation of other bioactive phytoconstituents from EP are being investigated in our lab presently.

## Abbreviations

AO: Acridine orange
DMSO: Dimethyl Sulphoxide
EB: Ethidium bromide
EP: *Elytranthe parasitica* (L.) Danser
EP.Aq: *Elytranthe parasitica* Aqueous fraction
EP.But: *Elytranthe parasitica* Butanol fraction
EP.DEE: *Elytranthe parasitica* Diethyl Ether fraction
EP.EA: *Elytranthe parasitica* Ethyl Acetate fraction
EP.M: *Elytranthe parasitica* methanol extract
EP.PE: *Elytranthe parasitica* Petroleum Ether fraction
GAE: Gallic acid Equivalent
HCC: Hepatocellular carcinoma
HPTLC: High Pressure Thin Layer Chromatography
IC50: Half maximal inhibitory concentration
QE: Quercetin Equivalent
Rf: Retention factor
ROS: Reactive Oxygen Species
SRB: Sulforhodamine B
TBARS: Thiobarbituric Acid Reactive Substance
TLC: Thin Layer Chromatography
